# 3D Printed Bioinspired Stents with Photothermal Effects for Malignant Colorectal Obstruction

**DOI:** 10.34133/2022/9825656

**Published:** 2022-07-01

**Authors:** Cheng Lin, Zhipeng Huang, Qinglong Wang, Wantao Wang, Wenbo Wang, Zhen Wang, Liwu Liu, Yanju Liu, Jinsong Leng

**Affiliations:** ^1^Centre for Composite Materials and Structures, Harbin Institute of Technology, No. 2 Yikuang Street, Harbin 150001, China; ^2^Tangdu Hospital of the Air Force Military Medical University, No. 1, Xinsi Road, Xi'an 710038, China; ^3^The First Affiliated Hospital of Harbin Medical University, No. 23 Youzheng Street, Nangang District, Harbin 150001, China; ^4^The Second Affiliated Hospital of Harbin Medical University, No. 246 Xuefu Street, Nangang District, Harbin 150001, China; ^5^Department of Astronautical Science and Mechanics, Harbin Institute of Technology, No. 92 West Dazhi Street, Harbin 150001, China

## Abstract

Stent placement is an effective palliation therapy for malignant colorectal obstruction. However, recurrent obstruction is a common severe complication caused by tumor ingrowth into the stent lumen. Conventional covered stents play a part in preventing the tumor from growing inward but at the expense of significantly increasing the risk of stent migration. Therefore, there is an urgent demand to develop stents with sustained antitumor and antimigration abilities. Herein, we propose a facile method for fabricating multifunctional bioinspired colorectal stents using 3D printing technology. Inspired by high-adhesion biological structures (gecko feet, tree frog toe pads, and octopus suckers) in nature, different types of bioinspired colorectal stents are designed to reduce migration. After functionalization with graphene oxide (GO), bioinspired colorectal stents show excellent and controllable photothermal performance, which is validated by effective ablation of colon cancer cells in vitro and tumors in vivo. Besides, the bioinspired colorectal stents demonstrate the feasibility of transanal placement and opening of the obstructed colon. More importantly, the facile manufacturing process of multifunctional bioinspired colorectal stents is appealing for mass production. Hence, the developed multifunctional bioinspired colorectal stents exhibit a highly promising potential in clinical applications.

## 1. Introduction

Colorectal cancer is the third most common cancer worldwide, with more than 1.8 million new cases diagnosed in 2018 (World Cancer Research Fund International). Approximately 7%-29% of patients with colorectal cancer suffer from acute colorectal obstruction, a life-threatening condition requiring urgent decompression [1]. If not treated effectively, the mortality rate can be as high as 80% to 90% within five years [2]. Because of the severe side effects and endless pain caused by traditional treatments of surgical removal and chemo/radiotherapy, stent placement is an efficient method to alleviate malignant obstruction in patients with advanced disease, extracolonic malignancy, high surgical risk, and poor general health [2]. Since the first report of self-expanding metal stents (SEMSs) by Dohmoto, SEMS has been widely used for malignant obstruction [3]. However, due to the lack of sustained antitumor ability, tumor growth into the lumen of the stent remains a serious problem, with a 100% probability of tumor occurrence in bare SEMS [4, 5]. Although conventional partially/fully covered SEMS developed based on bare SEMS can inhibit tumor ingrowth to some extent, unfortunately, tumors can still be found in 53% of patients with these stents [5]. More seriously, partially/fully covered stents lead to a high risk of stent migration, with an overall migration rate of up to 30% to 50% [6]. Hence, the development of multifunctional colorectal stents with antitumor and antimigration abilities is of great significance to meet the urgent demands of the treatment of malignant obstruction. However, the relevant work is missing in the literature, which is the impetus of this work.

Studies have shown that the surface microstructures of biological structures with high adhesion in nature (such as tree frog toe pads, octopus suckers, and gecko feet) play a vital role in preventing migration and enhancing adhesion [7–14]. Therefore, the introduction of these structures in stent design will effectively reduce migration, but the complex structures in nature far exceed the abilities of traditional manufacturing methods. Fortunately, the emergence of 3D printing (also known as additive manufacturing) has offered new opportunities for bionic manufacturing and has developed rapidly in the field of tissue engineering due to personalization and rapid prototyping [15–18]. However, there are few reports on the design and application of 3D printing in the treatment of colorectal cancer and malignant colorectal obstruction.

The goal of this work is to propose a facile method to develop multifunctional bioinspired colorectal stents with antimigration and antitumor abilities. In detail, bioinspired self-expandable colorectal stents that mimic octopus suckers, tree frog toe pads, and gecko feet were designed to restore the size of the intestinal lumen and reduce the possibility of migration. Polylactic acid (PLA)/polyurethane (PU)/drug (AUD) composite filaments were prepared, and the bioinspired stents were fabricated through 3D printing to ensure personalization and ideal matching [19–22]. Taking gentamycin sulfate (GS) as an example, a drug cumulative release study of bioinspired stents loaded with GS was carried out to confirm the feasibility of the stents for drug loading. Besides, graphene oxide (GO) was employed to functionalize the bioinspired stents, which can convert the absorbed near-infrared (NIR) light energy into heat energy at the tumor site, enabling ablation of the tumor with little effect on healthy tissue (the process is known as photothermal therapy) [23–30]. Due to high photothermal conversion efficiency, good cell compatibility, and no apparent toxicity in vivo, GO has been used in a wide range of biomedical applications such as drug/gene delivery carriers, biosensors, cell culture platforms, cancer treatment, and disease detection [23, 31–36]. In addition, the mechanical properties and antimigration ability of the bioinspired colorectal stents were systematically evaluated. The photothermal properties of the bioinspired colorectal stents were examined by ablation of colon cancer cells and tumor tissues in mice. Finally, the effectiveness of the bioinspired colorectal stents to expand the obstructed colon and the feasibility of transanal stent placement were verified.

## 2. Results and Discussion

**Figure 1:**
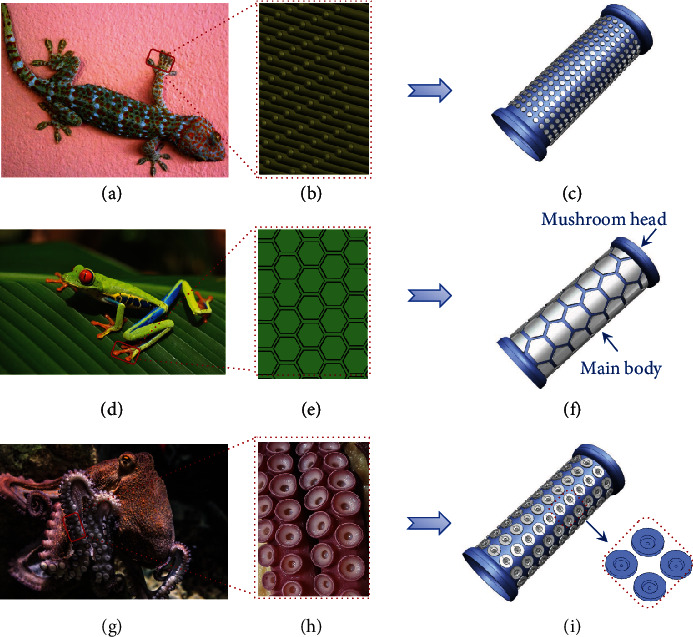
Design of bioinspired colorectal stents. (a) Gecko. (b) Schematic illustration of gecko foot setae. (c) Gecko-inspired colorectal stent. (d) Tree frog. (e) Schematic illustration of the hexagonal microstructure of the tree frog toe pad. (f) Tree frog-inspired colorectal stent. (g) Octopus. (h) Image of the octopus sucker. (i) Octopus-inspired colorectal stent.

Three types of bioinspired colorectal stents were developed to reduce migration based on highly adhesive biological structures in nature, including gecko feet, tree frog toe pads, and octopus suckers. These three biological structures have the following distinctive features to ensure high adhesion: the countless setae on the toes of the gecko's feet allow the gecko to traverse vertical surfaces at a speed of more than 1 m s^−1^ (Figures 1(a) and 1(b)) [12, 37]; the hexagonal microstructure separated by narrow passages on the tree frog toe pads ensures that the tree frog can adhere firmly during climbing (Figures 1(d) and 1(e)) [9, 38]; the octopus sucker, consisting of a hole and a dome-like protuberance, is fastened to the base through the void generated by its structural depression (Figures 1(g) and 1(h)) [13]. The bioinspired colorectal stents with setae-like, hexagonal, and octopus sucker-like microstructures on the surfaces are shown in Figures 1(c), 1(f), and 1(i), and the microstructure of each type of bioinspired stent is designed with three different heights (0.3 mm, 0.6 mm, and 1.2 mm). The stent is composed of the main body and the mushroom heads at both ends, where the mushroom heads are designed to further reduce migration. The design geometric parameters of bioinspired colorectal stents are detailed in Figure S1 and Table S1. For convenience, bioinspired colorectal stents with different microstructure heights inspired by geckos (G), tree frogs (T), and octopuses (O) are denoted as G0.3, G0.6, G1.2, T0.3, T0.6, T1.2, O0.3, O0.6, and O1.2, where the values represent the microstructure heights (mm).

**Figure 2:**
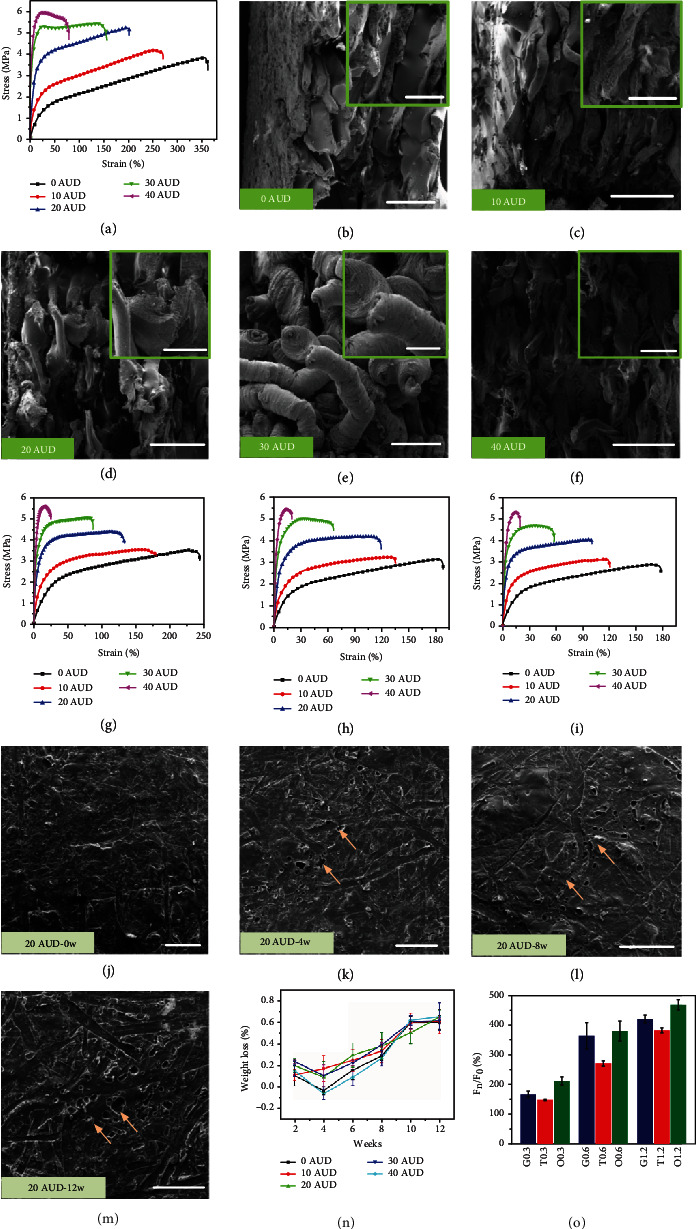
(a) Tensile testing of AUDs. Fracture morphology of AUD tensile specimens. (b) 0AUD, (c)10AUD, (d) 20AUD, (e) 30AUD, and (f) 40AUD. (b–f) Scale bar = 500 *μ*m/200 *μ*m (insets). Tensile testing of AUDs after different incubation weeks. (g) Four weeks, (h) 8 weeks, and (i) 12 weeks. Surface morphology of printed 20AUD specimens after different incubation weeks. (j) Zero weeks, (k) 4 weeks, (l) 8 weeks, and (m) 12 weeks. (j–m) Scale bar = 500 *μ*m. (n) Weight loss of AUDs after incubation with PBS. (o) Improvement of the antimigration ability of the bioinspired colorectal stents. *F*_*n*_: the antimigration force of bioinspired colorectal stents. *F*_0_: the antimigration force of colorectal stents with smooth surfaces (without bioinspired microstructures).

AUD composites with different compositions were prepared and coded as 0AUD, 10AUD, 20AUD, 30AUD, and 40AUD, where the numerical values indicate that the PLA content in the composites was 0, 10, 20, 30, and 40 wt.%, respectively. The mechanical properties of AUDs were investigated, as shown in Figure 2(a). With the increase of PLA content, the elongation decreased while the strength increased. The strengths of 20AUD, 30AUD, and 40AUD were approximately 5.2 MPa, 5.4 MPa, and 6.0 MPa. The elongation at break of 10AUD and 20AUD was up to 270% and 200%, respectively. Correspondingly, highly oriented stretched fibers were exhibited in the fracture morphology of AUDs, showing obvious ductile fracture features (Figures 2(b)–2(f)) [39, 40]. Compared with 10AUD, 20AUD, and 30AUD, the stretched fiber length of 40AUD was significantly shorter, which corresponded to the lowest elongation at break in Figure 2(a). In addition, the effect of the in vivo microenvironment on the mechanical properties of AUDs after implantation was simulated and studied in vitro by incubating the AUDs with phosphate buffered saline (PBS). After incubation for 4 weeks, the strength of AUDs changed slightly but the elongation at break decreased visibly (Figure 2(g)). The elongation at break of 0AUD, 10AUD, and 20AUD was reduced to approximately 250%, 180%, and 130%, respectively, which was attributed to the chain cleavage of AUDs [41]. The elongation at break decreased more in the first 4 weeks, decreased less in the next few weeks, and showed signs of gradual stabilization (Figures 2(a) and 2(g)–2(i)), which was consistent with the results in the literature [42]. It was worth noting that even after 12 weeks of incubation, the strength and elongation at break of 20AUD still exceeded 4.0 MPa and 100%, respectively (Figure 2(i)). In addition, the formation of holes on the surface of AUDs was observed (indicated by yellow arrows in Figures 2(k)–2(m)), which was due to the water absorption of the polymer matrix triggering degradation, resulting in the production of oligomers and monomers. When the degradation products accumulated and reached critical osmotic pressure, the surface was fractured and holes were formed. Oligomers and monomers were then released through the holes leading to weight loss, a process known as erosion [41, 43, 44]. After 12 weeks of incubation, the weight loss was between 0.61% and 0.66%, and the increase in weight after 4 weeks of incubation was because of water absorption (Figure 2(n)) [41].

**Figure 3:**
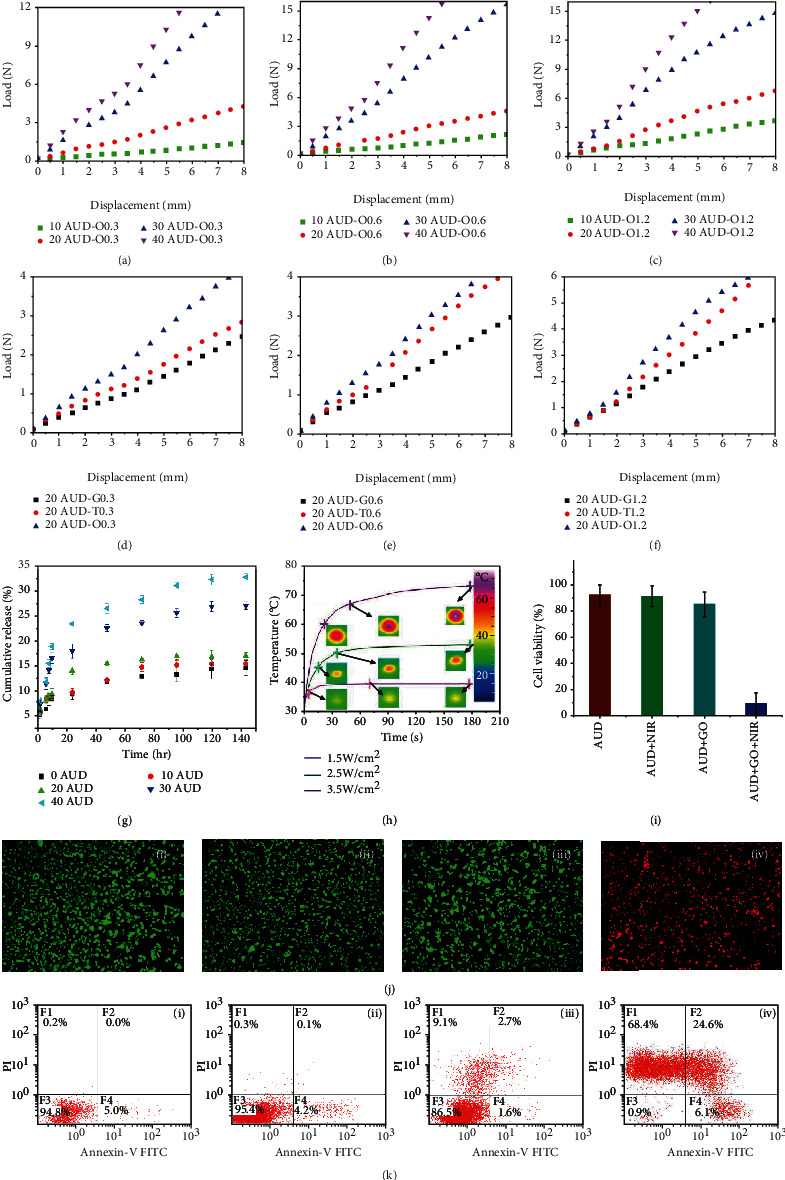
(a–f) Compressive behaviors of bioinspired colorectal stents. Octopus-inspired AUD colorectal stents with microstructure heights of (a) 0.3 mm, (b) 0.6 mm, and (c) 1.2 mm. Bioinspired 20AUD colorectal stents with different heights of surface microstructures. (d) 0.3 mm, (e) 0.6 mm, and (f) 1.2 mm. (g) Time-dependent cumulative release of GS in octopus-inspired AUD colorectal stents. (h) Temperature profiles at the irradiation sites of the bioinspired GO-O0.6-20AUD colorectal stents under various NIR power densities. (i) Cell viability of DLD-1 cancer cells after different treatments. (j) Live/dead assay of DLD-1 cancer cells after being treated with (i) AUD, (ii) AUD+NIR, (iii) AUD+GO, and (iv) AUD+GO+NIR. Living cells are shown in green and dead cells in red. Scale bar = 250 *μ*m. (k) Flow cytometric analysis of DLD-1 cancer cells after being treated with (i) AUD, (ii) AUD+NIR, (iii) AUD+GO, and (iv) AUD+GO+NIR. Quadrants F1, F2, F3, and F4 represent necrosis, advanced apoptosis, and alive and early apoptosis, respectively.

The antimigration ability of the bioinspired colorectal stents was evaluated by inserting the stents into the fresh swine colon and the same size colorectal stent with a smooth surface was used as the control group (Figure 2(o)). The antimigration ability of the colorectal stents increased with the height of the bioinspired microstructures and was greatly affected by the type of surface microstructure. The octopus-inspired colorectal stents exhibited the strongest antimigration ability, with O0.3, O0.6, and O1.2 showing 210%, 380%, and 470% improvement in antimigration ability compared with stents without bioinspired microstructures. This was because of the unusual anatomical structure of the octopus suckers, which were fastened to the substrate through the voids generated by their structural collapse, enabling the improvement of antimigration ability. In addition, the compressive behaviors of bioinspired colorectal stents were investigated since the bioinspired colorectal stent will be subjected to compression load from the intestinal wall and other biological tissues after implantation. As demonstrated in Figures 3(a)–3(c), the ability of the stents to resist compression loads increased with the increase of PLA content and the surface microstructure height. In addition, the type of surface microstructure also affected the ability to resist compression loads, with octopus-inspired colorectal stents showing the strongest ability, followed by tree frog-inspired colorectal stents, and finally by gecko-inspired colorectal stents (Figures 3(d)–3(f)). The compressive behaviors of bioinspired colorectal stents with different microstructure heights are illustrated in detail in Figure S2. The intuitive deformation process of the O0.6 20AUD colorectal stent with displacement and strain distributions is shown in Figure S3.

Besides, the drug-loading ability of the bioinspired colorectal stents was investigated and Figure 3(g) shows the cumulative release of GS over time. GS is a common aminoglycoside antibiotic that can reduce the number and virulence of intestinal bacteria by blocking bacterial protein synthesis, thus reducing the risk of infection in immunocompromised patients with malignant obstruction [45, 46]. The cumulative release ratio of AUD stents increased with the increase of PLA content, and the cumulative release ratio of 40AUD stents was about 33% at 144 h. In this work, GS was taken as an example to verify the feasibility of the developed multifunctional bioinspired stents as drug carriers. Other drugs, such as anticancer drugs, can also be loaded to enhance the therapeutic effect of the stents.

To examine the photothermal performance of bioinspired colorectal stents in vitro, a circular area with a diameter of approximately 10 mm on the GO functionalized AUD (GO-AUD) stent was randomly selected to be irradiated by NIR laser and the temperature was monitored. Based on the test results of mechanical properties and antimigration ability, O0.6-20AUD was selected as the optimal bioinspired stent for the following experiments. Figure 3(h) displays the temperature profiles at the irradiation sites of bioinspired GO-O0.6-20AUD stents under different NIR power densities, and the infrared thermographic photographs (corresponding to the points marked with crosses on the curves) are shown as insets. As the irradiation time increased, the temperature of the irradiation site increased rapidly in the initial stage and then gradually stabilized. The maximum temperature of the irradiation site was 39°C, 53°C, and 73°C, respectively, after 180 s of NIR laser irradiation with a power density of 1.5 W cm^−2^, 2.5 W cm^−2^, and 3.5 W cm^−2^. Therefore, the designed bioinspired colorectal stents possessed controllable excellent photothermal performance, which can be modulated by radiation duration and NIR power density.

Encouraged by the remarkable photothermal performance, the ability of the bioinspired colorectal stents to kill cancer cells was assessed. The DLD-1 colon cancer cells were treated with AUD, AUD+NIR, AUD+GO, and AUD+GO+NIR, respectively. As displayed in Figure 3(i), the cell viability of cancer cells treated with AUD, AUD+NIR, and AUD+GO was maintained at a high level. In contrast, the viability of cancer cells treated with AUD+GO+NIR was dramatically decreased, showing a remarkably improved anticancer effect. The viability status of cancer cells was visualized by live/dead assay, and the significant comparison between the four groups with and without photothermal therapy is shown in Figure 3(j). The cancer cells in the AUD+GO+NIR group exhibited intense red fluorescence, indicating a large number of dead cells. Comparatively, the cancer cells in the other three groups presented strong green fluorescence, demonstrating that most cells were alive. Similar results were also verified by flow cytometric analysis, which showed that the apoptosis and necrosis rates of cells treated with AUD+GO+NIR were significantly higher than those treated with AUD, AUD+NIR, and AUD+GO (Figure 3(k)). Hence, it can be concluded that the bioinspired colorectal stents can effectively kill DLD-1 colon cancer cells under NIR irradiation.

**Figure 4:**
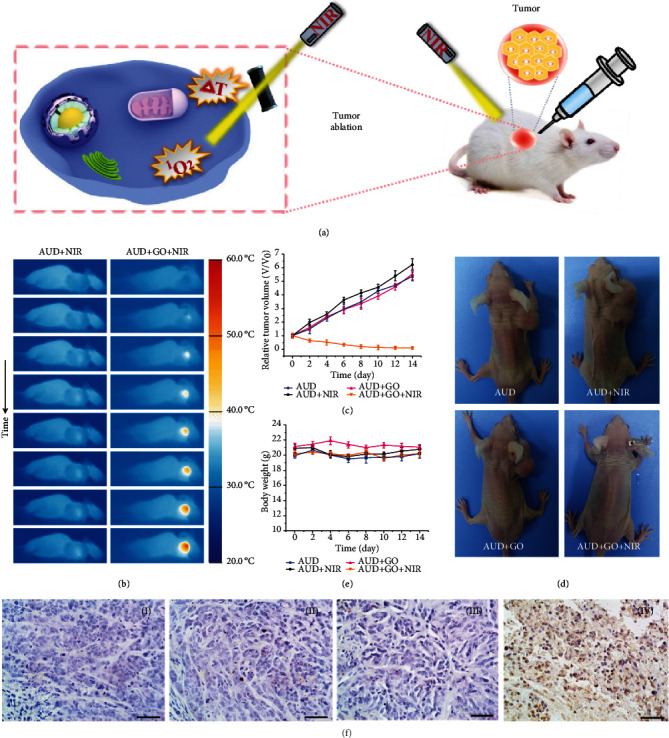
(a) Schematic illustration of photothermal therapy in tumor-bearing mice. (b) Infrared thermal images of tumor-bearing mice treated with AUD+NIR and AUD+GO+NIR. (c) Tumor growth profiles of the mice with different treatments. (d) Photographs of tumor-bearing mice after different treatments on day 15. (e) Bodyweight curves of mice with different treatments. (f) TUNEL stained images of tumor tissues on day 15 after being treated with (i) AUD, (ii) AUD+NIR, (iii) AUD+GO, and (iv) AUD+GO+NIR. Scale bar = 50 *μ*m.

The effectiveness of ablating tumor cells in vitro prompted us to further evaluate the antitumor efficacy of the bioinspired colorectal stents in vivo (Figure 4(a)). Tumor-bearing mice were selected and randomized into four treatment groups: (1) AUD, (2) AUD+NIR, (3) AUD+GO, and (4) AUD+GO+NIR. Figure 4(b) displays the infrared thermal images of tumor-bearing mice treated with AUD+NIR and AUD+GO+NIR. It can be observed that there was no noticeable temperature increase in the AUD+NIR group, while the maximum temperature of the tumor site in the AUD+GO+NIR group increased to more than 50°C under laser irradiation. This hyperthermia at the tumor site was effective for tumor ablation because it caused functional damage to the cell membrane, mitochondria, DNA, and RNA of the tumor cells [24]. As a result, after different treatments, the tumor volume of mice reduced obviously in the AUD+GO+NIR group, whereas that of mice in the other three groups increased rapidly (Figures 4(c)–4(e)). The outstanding outcomes of photothermal therapy also showed the stability of GO in vivo. Besides, the weight fluctuation of tumor-bearing mice in each group was negligible, demonstrating the biocompatibility of AUD and GO-AUD (Figure 4(e)). In addition, terminal deoxynucleotidyl transferase-mediated dUTP-biotin nick end labeling (TUNEL) stained images of tumors demonstrated the antitumor efficiency of the AUD+GO+NIR group was significantly better than the other three groups, exhibiting a cell necrosis rate higher than 95% (Figure 4(f)).

**Figure 5:**
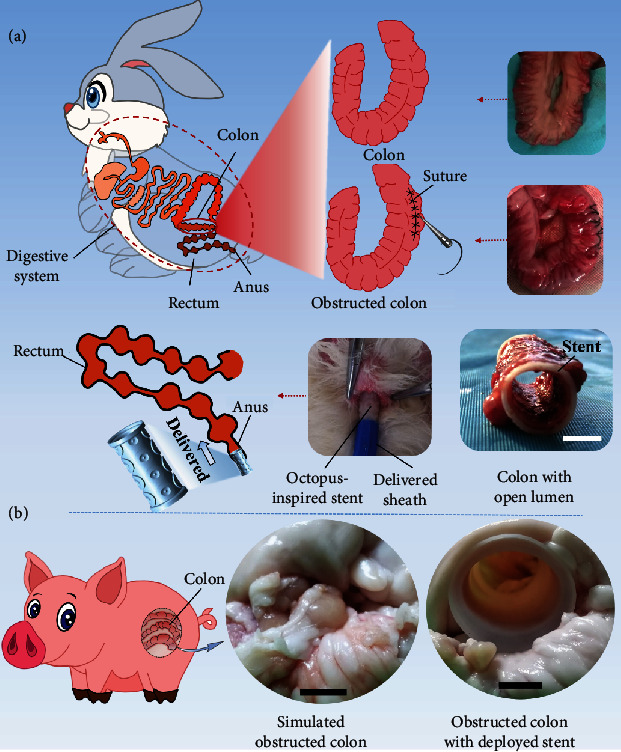
Feasibility of the bioinspired colorectal stent for transanal placement opening of the obstructed colon. (a) Scale bar = 5 mm; (b) scale bar = 10 mm.

In addition, the bioinspired colorectal stent demonstrated the feasibility of transanal placement and opening of the obstructed colon (Figure 5). Transanal stent placement is an effective method for palliating obstruction, with the advantages of high efficiency and few complications. An obstructed rabbit colon with reduced lumen diameter was obtained by suturing part of the colon. The compressed bioinspired stent was delivered through a catheter and entered the colon via the anus. Once the target position was determined, the bioinspired stent was pushed out of the catheter and a gradual self-expansion process was automatically carried out, verifying the self-expanding ability of the bioinspired stent. Figure 5(a) shows the transanal placement process and the rabbit colon with an open lumen. The effectiveness of the bioinspired colorectal stent to expand the obstructed swine colon was also verified due to the similarity in size and configuration between the swine colon and the human colon. After the stent was implanted into the simulated obstructed swine colon, the stent can self-deploy and the colon with a deployed stent was able to restore its open lumen (Figure 5(b)).

## 3. Conclusion

In summary, we proposed a facile and effective strategy to successfully integrate antitumor, antimigration, and drug-loading abilities into a single 3D printed bioinspired colorectal stent. Compared with previous studies, the advancement of this work lays in (1) the functionalized nonmesh bioinspired stent minimized the risk of tumor growth into the lumen of the stent. It not only physically prevented tumor inward growth but also ensured complete ablation of the tumor through prominent photothermal performance. (2) The stent with bioinspired surface microstructures greatly improved the antimigration force of the stent and reduced the probability of stent migration. The antimigration force of the bioinspired stent was increased by up to 470% compared to the stent without surface microstructure. Braided mesh stents in previous work increased the risk of tumor growth into the lumen of the stent and increase the tendency for reobstruction, which may require a second operation, although membrane-covered stents can prevent tumor growth inward to a certain extent, at the expense of a greatly increased probability of stent migration. (3) In addition, compared with the traditional braiding method, the bioinspired stent was prepared by additive manufacturing, which allowed for the customization of the stent and the implementation of precision medicine, thus avoiding tissue wear and other complications caused by the mismatch.

All the results clearly demonstrate the multifunctional bioinspired colorectal stent is a promising candidate for the treatment of malignant colorectal obstruction. Besides, it is worth noting that the manufacturing process of the multifunctional bioinspired colorectal stents does not involve any complex reaction fluid, which is attractive for large-scale production of clinical applications. In addition, the design and preparation approach of stents is in line with the zeitgeist of personalized medicine and is expected to show broad application potential.

## 4. Materials and Methods

### 4.1. Preparation of AUD Composite Filaments

PLA pellets (Nature Works LLC.), PU pellets (BASF Polyurethanes GmbH), and GS powders (Macklin Biochemical Co. Ltd.) were physically mixed by a mixer. The PLA and PU pellets were dried at 45°C in a vacuum chamber for 12 h before extruding. The mixture was then melt-blended and extruded using a corotating twin screw extruder with a nozzle diameter of 1.75 mm, an extrusion speed of 50 rpm, and an extrusion temperature of 180°C-210°C (different heating chambers). Four different AUD composites were prepared with the mass fraction of PLA/PU as 10/90, 20/80, 30/70, and 40/60, among which the mass fraction of drug (GS) was 4 wt. %. According to the mass fraction of PLA, the four AUD composites were abbreviated as 10AUD, 20AUD, 30AUD, and 40AUD, and pure PU was denoted as 0AUD as the control.

### 4.2. Preparation of GO Functionalized Bioinspired Colorectal Stents

The models of the bioinspired colorectal stents were built by UG NX 10.0, and the geometric parameters of the stents were determined by referring to the parameters of commercially available colorectal stents (MICRO_TECH (Nanjing) Co., Ltd). The stents were printed via an FDM 3D printer (Allcct, Wuhan, China) with printing speed, layer height, and nozzle diameter of 3 mm s^−1^, 0.1 mm, and 0.4 mm. GO and distilled water were mixed proportionally and placed in an ultrasonic bath at room temperature for 240 min to obtain a uniform GO suspension (1 mg ml^−1^). Then, GO suspension was evenly coated on the surface of the stent and dried thoroughly in the oven at 50°C. The process was repeated three times to obtain the GO functionalized bioinspired colorectal stents.

### 4.3. Mechanical Tests

Tensile test specimens were printed and subjected to uniaxial tensile tests following ASTM D638 until failure. Compression tests of colorectal stents were carried out according to ASTM D2412 [47]. All tests were performed through a Zwick 010 tester with a test temperature of 37°C and a loading rate of 2 mm min^−1^. The specimens were heated from room temperature (25°C) to 37°C at a heating rate of 3°C min^−1^ and then maintained for 15 min. Three specimens of each type were measured, and the average results were exported. The VEGA3 TESCAN scanning electron microscope was operated at a voltage of 10 kV, and the fracture morphology was observed.

### 4.4. In Vitro Biodegradation

The printed tensile test specimens were incubated with phosphate buffered saline (PBS, pH = 7.4) in a thermo-stated shaking water bath at 37°C with 100 rpm rotation. The solution was replaced every week. Specimens were periodically taken out, rinsed with distilled water, and dried to a constant weight in a vacuum. The weight of specimens was recorded every two weeks, and the weight loss ratio (%) was obtained according to the following:

(1) $$\begin{array}{c} \text{Weight loss }\left(\%\right)=-\frac{w_{t}-w_{0}}{w_{0}}\times 100\%, \end{array}$$

where *w*_0_ is the initial weight of the specimen before incubation and *w*_*t*_ is the weight of the specimen at time *t*.

### 4.5. Antimigration Tests

The antimigration ability of the bioinspired colorectal stents was tested using fresh swine colons at 37°C. First, the colon was sutured axially to obtain a colon with 20% obstruction in the radial direction. Then, the printed stent was inserted into the obstructed colon to obtain a reopened lumen. Next, the stent was connected to the lower fixture of the tester, and the colon was connected to the upper fixture of the tester. The upper fixture (colon) was moved upward at a loading rate of 1 mm min^−1^ until it was completely separated from the lower fixture (stent). The maximum force required for complete separation of the stent and colon was recorded as the antimigration force. The antimigration force of bioinspired colorectal stents was denoted as *F*_*n*_, and the antimigration force of colorectal stents with smooth surfaces (without bioinspired microstructures) was denoted as *F*_0_.

### 4.6. Cumulative Drug Release

O0.6-AUD stents were placed in a release medium (60 ml PBS, pH = 7.4) to investigate the cumulative drug release in the shaking water bath at 37°C and 100 rpm. The supernatant was taken out and analyzed at 1, 2, 6, 8, 10, 24, 48, 72, 96, 120, and 144 h, and the same amount of fresh PBS solution was added back to the release medium at each time point. The concentration of GS was measured with an ultraviolet (UV) spectrophotometer at a wavelength of 232 nm (*n* = 3). The cumulative release of GS was obtained by the following:

(2) $$\begin{array}{c} \text{Cumulative release }\left(\%\right)=\frac{C_{t}}{C_{\infty }}\times 100\%, \end{array}$$

where *C*_*t*_ is the amount of GS released at time t and *C*_∞_ is the maximum release amount of GS.

### 4.7. In Vitro Photothermal Performance

GO-O0.6-20AUD stents were irradiated with an 808 nm NIR laser (Changchun Laser Technology Co., Ltd) for 180 s at power densities of 1.5 W·cm^−2^, 2.5 W·cm^−2^, and 3.5 W·cm^−2^. A circular area with a diameter of approximately 10 mm on the stent was randomly selected to be irradiated by the NIR laser. The temperature evolution of the irradiated area was recorded by an infrared thermal imaging camera and analyzed by the FLIR R&D software.

### 4.8. Cell Viability Assay of Cancer Cells after Different Treatments

DLD-1 human colon cancer cells were used to evaluate the cell viability after four different treatments, including (1) O0.6-20AUD culture samples (AUD), (2) O0.6-20AUD culture samples+NIR laser (AUD+NIR), (3) GO-O0.6-20AUD culture samples (AUD+GO), and (4) GO-O0.6-20AUD culture samples+NIR laser (AUD+GO+NIR). O0.6-20AUD and GO-O0.6-20AUD stents were cut along the axis and expanded into a plane (excluding mushroom heads), from which samples with a diameter of 8 mm were randomly cut for cell culture. The DLD-1 cancer cells (4.0 × 10^5^ cells/well) were cultured in DMEM containing 10% fetal bovine serum (FBS) and 1% penicillin/streptomycin in an incubator at 5% CO_2_ and 37°C. After 1 d of culture, sterilized O0.6-20AUD and GO-O0.6-20AUD culture samples were gently placed into each well. After another 12 h of culture, the cells in AUD+NIR and AUD+GO+NIR groups were exposed to an 808 nm laser with a power density of 3.5 W cm^−2^ for 15 min. Then, the culture medium was removed, and the cell viability was evaluated by MTT assay. In detail, 50 *μ*l MTT (5 mg ml^−1^) was added to each well, cultured for 4 h, and solubilized with 250 *μ*l dimethyl sulfoxide (DMSO). 100 *μ*l DMSO was collected from each well and added to a 96-well plate. The absorbance value at 590 nm was recorded by a microplate reader. Cell viability was calculated by the following:

(3) $$\begin{array}{c} \text{Cell viability }\left(\%\right)=\frac{{\text{OD}}_{\text{AUDs}}-{\text{OD}}_{\text{control}}}{{\text{OD}}_{\text{control}}}\times 100\%, \end{array}$$

where the OD_AUDs_ were the absorbance at 590 nm after different treatments and OD_control_ was the absorbance at 590 nm in the control group. In addition, the cells were stained with calcein-AM and PI solution for live/dead staining (calcein-AM/PI, Dojindo) and observed by an inverted optical microscope (Leica, Germany). Besides, DLD-1 cancer cells in four groups were stained with Annexin-V-FITC and PI and analyzed by flow cytometer (Beckman Coulter, Brea, CA, USA) [48].

### 4.9. In Vivo Photothermal Therapy

Animal experiments were conducted under the protocol approved by the Ethics Committee of the First Affiliated Hospital of Harbin Medical University. Male Balb/c nude mice were provided by the First Affiliated Hospital of Harbin Medical University. The DLD-1 cancer cells suspended in PBS were subcutaneously injected into the right shoulder of each mouse. When the tumor volume reached about 270 mm^3^, the in vivo photothermal therapy was performed. Considering the volume of nude mice, the GO-O0.6-20AUD (abbreviated as GO-AUD) stents were cut along the axis and expanded into a plane, from which samples with a diameter of 10 mm were cut for photothermal therapy. Similarly, photothermal samples of O0.6-20AUD (abbreviated as AUD) with a diameter of 10 mm were cut from the O0.6-20AUD stents for the control groups. Twenty tumor-bearing mice were divided into four groups randomly (*n* = 5): (1) implanted with AUD samples (AUD), (2) implanted with AUD samples+NIR laser (AUD+NIR), (3) implanted with GO-AUD samples (AUD+GO), and (4) implanted with GO-AUD samples+NIR laser (AUD+GO+NIR). The sterilized AUD and GO-AUD photothermal samples were surgically implanted in the center of the tumor tissue. Tumor-bearing mice in AUD+NIR and AUD+GO+NIR groups were irradiated with 808 nm NIR at a power density of 4.5 W cm^−2^ for 10 min. The temperature elevation of the tumor site was measured by an infrared thermal camera. The tumor volume was calculated by the following:tumor volume (*V*) = (tumor length) × (tumor width)2/2 − photothermal sample volume; photothermal sample volume = sample surface area × sample height. The relative tumor size was defined as *V*/*V*_0_, where *V*_0_ was the tumor volume before treatment and *V* was the tumor volume after t days of treatment. The length and width of the tumor were measured using a digital caliper every 2 d. The tumor-bearing mice were euthanized on day 15 after the implantation of photothermal samples. The tumor tissues were harvested, immersed in 10% formalin, paraffin-embedded, and sectioned for TUNEL staining [49].

### 4.10. Feasibility of Transanal Placement and Opening of the Obstructed Colon

Healthy New Zealand white rabbits weighing 3.0~4.0 kg without gender restriction were provided by the First Affiliated Hospital of Harbin Medical University. The liquid diet was given 12-24 h and fasting within 12 h before the procedure. The rabbits were anesthetized by intravenous injection of pentobarbital sodium (1 ml kg^−1^), and then, the abdomen was shaved for preoperative skin preparation and disinfection. A small incision (3-4 cm) was made in the abdomen to obtain the colon, and the colon was sutured approximately 3 mm from the lateral line to obtain an obstructed colon with reduced lumen diameter. The bioinspired colorectal stent with a diameter of 9 mm and a length of 25 mm was loaded through a 26 French delivery catheter and then entered the colon via the anus to reach the obstruction site. The stent was slowly pushed out and released at the position of 2 cm beyond the distal end of the colon obstruction, ensuring that the stent covered all the obstructed segments. Besides, the bioinspired colorectal stents were inserted into the simulated obstructed swine colons to verify the ability to restore the open lumen of colons, which were obtained from adult swine models.

## Data Availability

All data is available in the main text or the supplementary materials.

## Supplementary Materials

Supplementary Materials Figure S1: (a–h) Design parameters of bioinspired colorectal stents. (a) Unfolded plane geometry of the main body of the gecko-inspired colorectal stent. (b) Setae-like microstructure on the surface of the gecko-inspired colorectal stent. (c) Side and top views of the gecko-inspired colorectal stent. (d) Unfolded plane geometry of the main body of the tree frog-inspired colorectal stent. (e) Hexagonal microstructure on the surface of tree frog-inspired colorectal stent. (f) Unfolded plane geometry of the main body of the octopus-inspired colorectal stent. (g) Octopus sucker-like microstructure on the surface of the octopus-inspired colorectal stent. (h) Structural decomposition of octopus sucker-like microstructure. (i) Fabrication of 3D printed bioinspired colorectal stents. Scale bar = 10 mm. Figure S2: compressive behaviors of bioinspired colorectal stents with different microstructure heights. (a) Gecko-inspired, (b) tree frog-inspired, and (c) octopus-inspired 10AUD colorectal stents. (d) Gecko-inspired, (e) tree frog-inspired, and (f) octopus-inspired 20AUD colorectal stents. (g) Gecko-inspired, (h) tree frog-inspired, and (i) octopus-inspired 30AUD colorectal stents. (j) Gecko-inspired, (k) tree frog-inspired, and (l) octopus-inspired 40AUD colorectal stents. Figure S3: compression deformation process of O0.6-20AUD colorectal stents (a) Experiments, (b) displacement distribution, and (c) strain distribution obtained by finite element analysis. Table S1: geometric parameters of bioinspired colorectal stents.
