# Supplementary material for: 3D Printed Bioinspired Stents with Photothermal Effects for Malignant Colorectal Obstruction
Source: Research (Wash D C). 2022 Jul 1;2022:9825656. doi: 10.34133/2022/9825656 (PMC9285633; doi:10.34133/2022/9825656)
Supplement: Supplementary Materials — Figure S1: (a–h) Design parameters of bioinspired colorectal stents. (a) Unfolded plane geometry of the main body of the gecko-inspired colorectal stent. (b) Setae-like microstructure on the surface of the gecko-inspired colorectal stent. (c) Side and top views of the gecko-inspired colorectal stent. (d) Unfolded plane geometry of the main body of the tree frog-inspired colorectal stent. (e) Hexagonal microstructure on the surface of tree frog-inspired colorectal stent. (f) Unfolded plane geometry of the main body of the octopus-inspired colorectal stent. (g) Octopus sucker-like microstructure on the surface of the octopus-inspired colorectal stent. (h) Structural decomposition of octopus sucker-like microstructure. (i) Fabrication of 3D printed bioinspired colorectal stents. Scale bar = 10 mm. Figure S2: compressive behaviors of bioinspired colorectal stents with different microstructure heights. (a) Gecko-inspired, (b) tree frog-inspired, and (c) octopus-inspired 10AUD colorectal stents. (d) Gecko-inspired, (e) tree frog-inspired, and (f) octopus-inspired 20AUD colorectal stents. (g) Gecko-inspired, (h) tree frog-inspired, and (i) octopus-inspired 30AUD colorectal stents. (j) Gecko-inspired, (k) tree frog-inspired, and (l) octopus-inspired 40AUD colorectal stents. Figure S3: compression deformation process of O0.6-20AUD colorectal stents (a) Experiments, (b) displacement distribution, and (c) strain distribution obtained by finite element analysis. Table S1: geometric parameters of bioinspired colorectal stents. [file 9825656.f1.pdf]

Research
A SCIENCE PARTNER JOURNAL
SPJ
SCIENCE PARTNER JOURNALS

Supplementary Materials

3D Printed Bioinspired Stents with Photothermal Effects for Malignant Colorectal Obstruction

Cheng Lin[1], Zhipeng Huang[3], Qinglong Wang[4], Wantao Wang[4], Wenbo Wang[4], Zhen Wang[5], Liwu Liu[2,*], Yanju Liu[2], Jinsong Leng[1,*]

Prof. Jinsong Leng, Cheng Lin
[1]Centre for Composite Materials and Structures, Harbin Institute of Technology, No. 2 Yikuang Street, Harbin 150001, P. R. China
Email: lengjs@hit.edu.cn

Prof. Liwu Liu, Prof. Yanju Liu
[2]Department of Astronautical Science and Mechanics, Harbin Institute of Technology, No. 92 West Dazhi Street, Harbin 150001, P. R. China
Email: liulw@hit.edu.cn

Zhipeng Huang,
[3]Tangdu Hospital of the Air Force Military Medical University, No. 1, Xinsi Road, Xi'an 710038, P. R. China

Qinglong Wang, Wantao Wang, Prof. Wenbo Wang,
[4]The First Affiliated Hospital of Harbin Medical University, No. 23 Youzheng Street, Nangang District, Harbin 150001, P. R. China

Prof. Zhen Wang
[5]The Second Affiliated Hospital of Harbin Medical University, No. 246 Xuefu Street, Nangang District, Harbin 150001, P. R. China

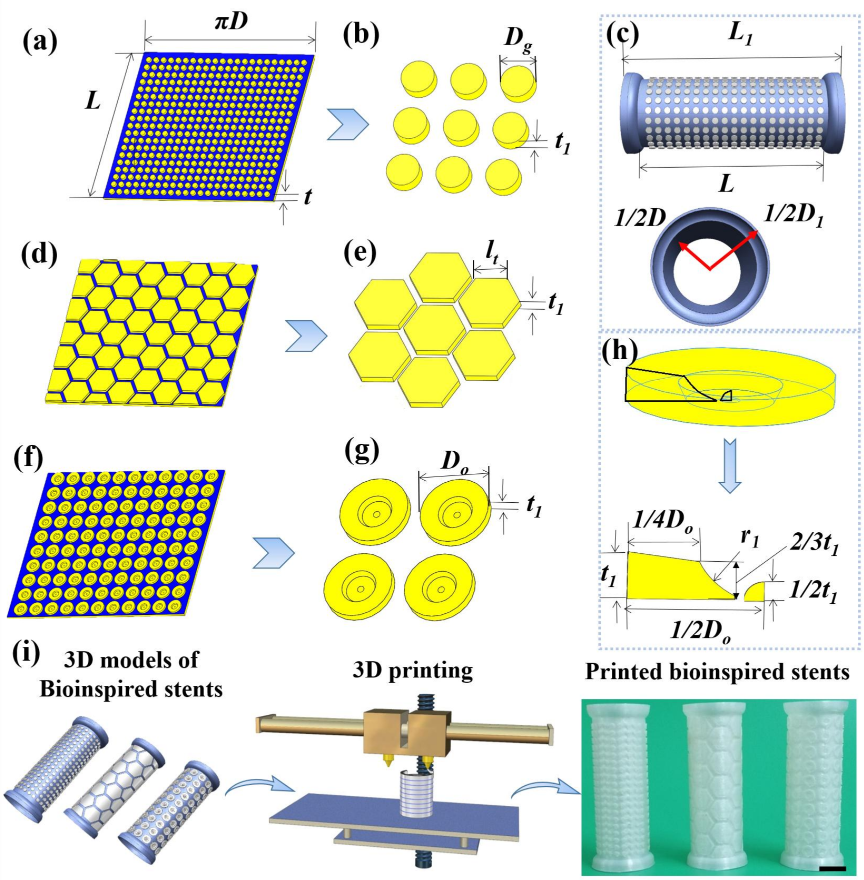

Figure S1. (a-h) Design parameters of bioinspired colorectal stents. (a) Unfolded plane geometry of the main body of the gecko-inspired colorectal stent. (b) Setae-like microstructure on the surface of the gecko-inspired colorectal stent. (c) Side and top views of the gecko-inspired colorectal stent. (d) Unfolded plane geometry of the main body of the tree frog-inspired colorectal stent. (e) Hexagonal microstructure on the surface of tree frog-inspired colorectal stent. (f) Unfolded plane geometry of the main body of the octopus-inspired colorectal stent. (g) Octopus sucker-like microstructure on the surface of the octopus-inspired colorectal stent. (h) Structural decomposition of octopus sucker-like microstructure. (i) Fabrication of 3D printed bioinspired colorectal stents. Scale bar = 10 mm.

Table S1. Geometric parameters of bioinspired colorectal stents.

Parameters	Values [mm]	Parameters	Values [mm]
D	22	D_g	2
L	60	t_1	0.3/0.6/1.2
t	0.45	l_t	5
L_1	70	D_o	5
D_1	28	r_1	1.8

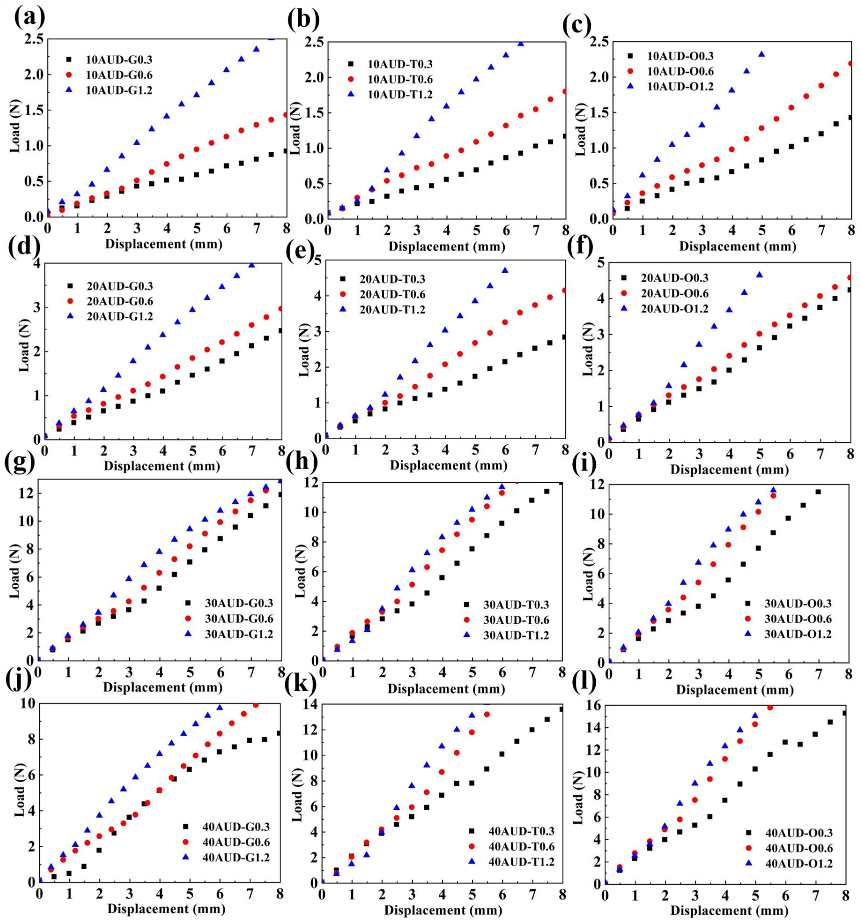

Figure S2. Compressive behaviors of bioinspired colorectal stents with different microstructure heights. (a) Gecko-inspired, (b) tree frog-inspired and (c) octopus-inspired 10AUD colorectal stents. (d) Gecko-inspired, (e) tree frog-inspired and (f) octopus-inspired 20AUD colorectal stents. (g) Gecko-inspired, (h) tree frog-inspired and (i) octopus-inspired 30AUD colorectal stents. (j) Gecko-inspired, (k) tree frog-inspired and (l) octopus-inspired 40AUD colorectal stents.

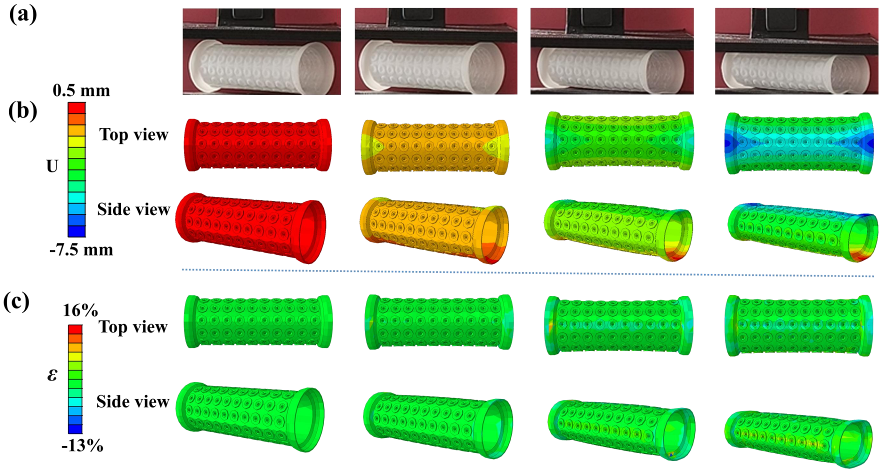

Figure S3. Compression deformation process of O0.6-20AUD colorectal stents (a) Experiments. (b) Displacement distribution and (c) strain distribution obtained by finite element analysis.
